# Synthesis, optical and electrochemical properties of (D–π)_2_-type and (D–π)_2_Ph-type fluorescent dyes

**DOI:** 10.3762/bjoc.18.106

**Published:** 2022-08-18

**Authors:** Kosuke Takemura, Kazuki Ohira, Taiki Higashino, Keiichi Imato, Yousuke Ooyama

**Affiliations:** 1 Applied Chemistry Program, Graduate School of Advanced Science and Engineering, Hiroshima University, 1-4-1 Kagamiyama, Higashi-Hiroshima 739-8527, Japan

**Keywords:** (D–π)_2_ structure, fluorescence, fluorescent dyes, photoabsorption, redox properties

## Abstract

The (D–π)_2_-type fluorescent dye **OTT-2** with two (diphenylamino)carbazole-thiophene units as D (electron-donating group)–π (π-conjugated bridge) moiety and the (D–π)_2_Ph-type fluorescent dye **OTK-2** with the two D–π moieties connected through a phenyl ring were derived by oxidative homocoupling of a stannyl D–π unit and Stille coupling of a stannyl D–π unit with 1,3-diiodobenzene, respectively. Their optical and electrochemical properties were investigated by photoabsorption and fluorescence spectroscopy, time-resolved fluorescence spectroscopy, cyclic voltammetry (CV) and molecular orbital (MO) calculations. In toluene the photoabsorption and fluorescence maximum wavelengths (λ_max,abs_ and λ_max,fl_) of **OTT-2** appear in a longer wavelength region than those of **OTK-2**. The fluorescence quantum yield (Φ_fl_) of **OTT-2** is 0.41, which is higher than that (Φ_fl_ = 0.36) of **OTK-2**. In the solid state **OTT-2** shows relatively intense fluorescence properties (Φ_fl-solid_ = 0.24 nm), compared with **OTK-2** (Φ_fl-solid_ = 0.15 nm). CV results demonstrated that **OTT-2** and **OTK-2** exhibit a reversible oxidation wave. Based on photoabsorption, fluorescence spectroscopy and CV for the two dyes, it was found that the lowest unoccupied molecular orbital (LUMO) energy level of **OTT-2** is lower than that of **OTK-2**, but **OTT-2** and **OTK-2** have comparable highest occupied molecular orbital (HOMO) energy levels. Consequently, this work reveals that compared to the (D–π)_2_Ph-type structure, the (D–π)_2_-type structure exhibits not only a bathochromic shift of the photoabsorption band, but also intense fluorescence emission both in solution and the solid state.

## Introduction

The design and development of a new type of organic fluorescent dyes have been of considerable scientific and practical concern with the objective of not only fundamental studies [1–13] in synthetic chemistry, electrochemistry and photochemistry, but also their potential applications to emitters for optoelectronic devices, such as organic light-emitting diodes (OLEDs) [14–22], as well as fluorescent probes [23–28] for bioimaging and fluorescent sensors for specific target species [29–32]. Among many kinds of organic fluorescent dyes, much efforts have been made on the development of donor–π–acceptor (D–π–A)-type fluorescent dyes constructed of an electron-donating moiety (D) and an electron-withdrawing moiety (A), linked by a π-conjugated unit thanks to their intense photoabsorption and fluorescence emission characteristics originating from the intramolecular charge transfer (ICT) excitation from the D to the A moiety [4–9,18–20,25–26]. Furthermore, the (D–π–)_2_A-type fluorescent dyes with two D–π moieties have recently been stimulating intensive research efforts because of their high molar extinction coefficients and fluorescence quantum yields, compared to those of D–π–A-type fluorescent dyes [10–13,21–22,27–28,32].

**Scheme 1:**
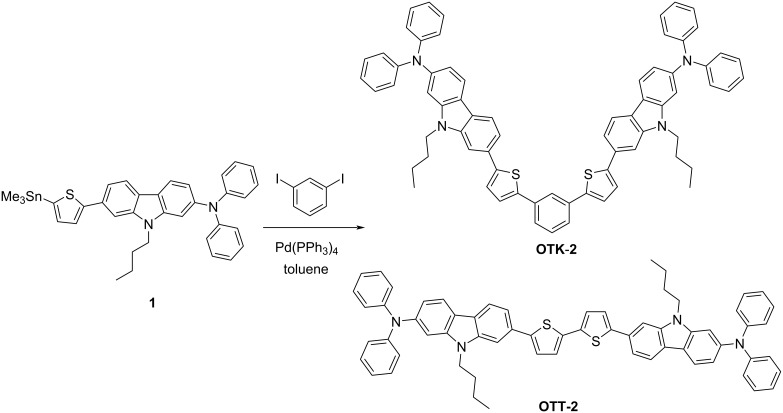
Synthesis of **OTK-2** and **OTT-2**.

In our previous work [33], we have reported the synthesis, optical and electrochemical properties of the (D–π)_2_Ph-type fluorescent dye **OTK-2** with two (diphenylamino)carbazole-thiophene units as D–π moiety connected through a phenyl ring (Scheme 1). The ICT-based photoabsorption and fluorescence bands of **OTK-2** appear in a shorter wavelength region than those of the corresponding (D–π)_2_A-type fluorescent dye having an azine ring (pyridine, pyrazine or triazine ring) as a substitute for the phenyl ring. However, the molar extinction coefficient (ε_max_) and fluorescence quantum yield (Φ_fl_) of **OTK-2** are comparable to those of the (D–π)_2_A-type fluorescent dyes. More recently, we found that the (D–π)_2_-type fluorescent dye **OTT-2** consisting of two D–π moieties is derived by oxidative homocoupling of a stannyl D–π unit. There is an obvious structural difference between the two dyes: **OTK-2** has a cross-conjugated system due to the involvement of the 1,3-phenylene unit as an additional linker, but **OTT-2** has a conjugated system. Therefore, it is interesting to reveal the optical and electrochemical properties of (D–π)_2_-type fluorescent dyes, making a comparison with (D–π)_2_Ph-type fluorescent dyes. Herein, we report the syntheses of (D–π)_2_-type and (D–π)_2_Ph-type fluorescent dyes and their optical and electrochemical properties based on photoabsorption and fluorescence spectroscopy, time-resolved fluorescence spectroscopy, cyclic voltammetry (CV) and molecular orbital (MO) calculations.

## Results and Discussion

Using a toluene solution containing 1,3-diiodobenzene and (diphenylamino)carbazole-thiophenestannane derivative **1** [33] in the presence of Pd(PPh_3_)_4_, the (D–π)_2_-type and (D–π)_2_Ph-type fluorescent dyes **OTK-2** [33] and **OTT-2** were obtained by Stille coupling of **1** with 1,3-diiodobenzene and oxidative homocoupling of **1**, respectively (Scheme 1).

**Figure 1:**
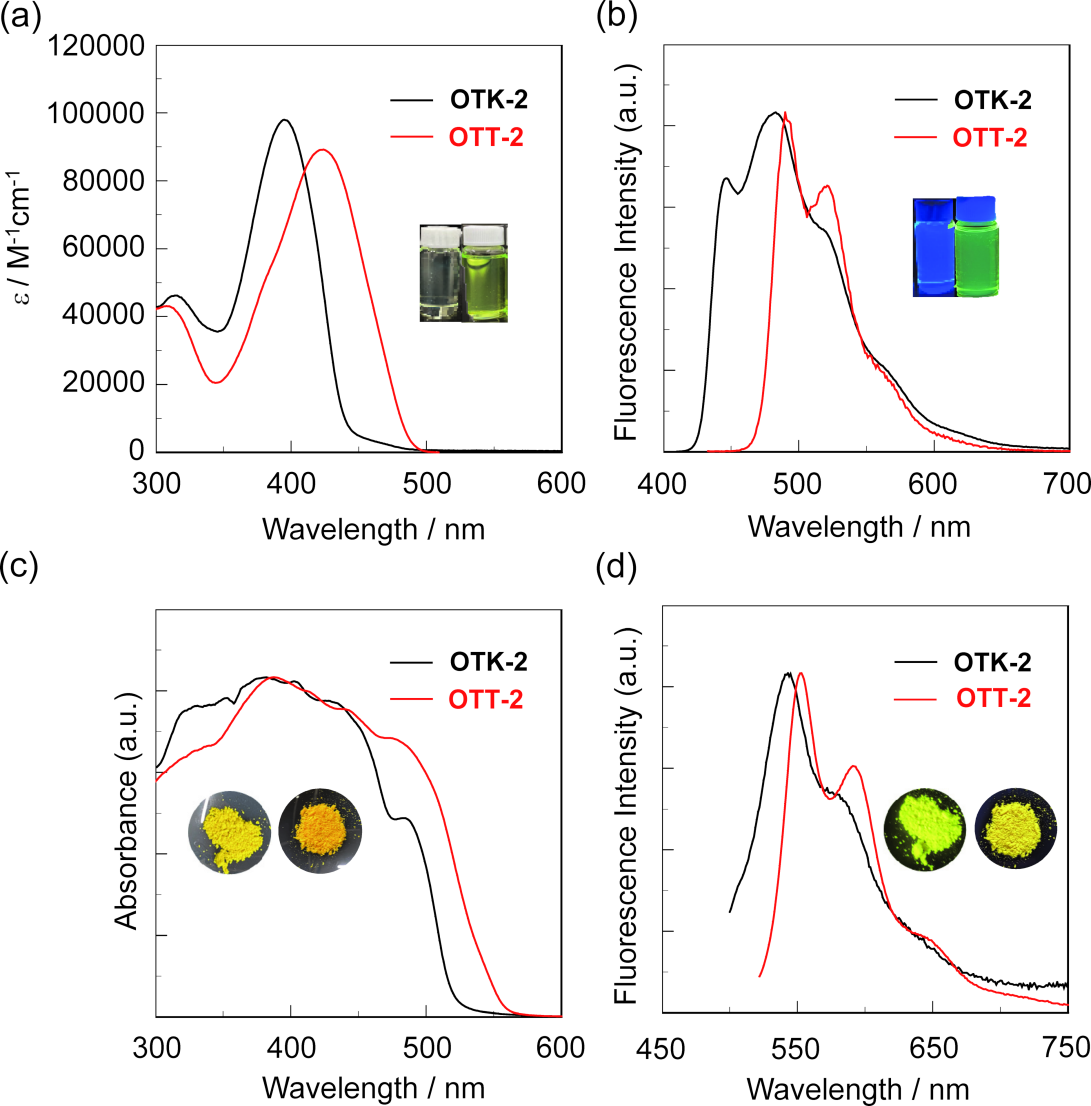
(a) Photoabsorption and (b) fluorescence (λ_ex_ = λ_max,abs_) spectra of **OTK-2** [33] and **OTT-2** in toluene. (c) Solid-state UV–vis diffuse reflection–absorption and (b) and (d) fluorescence spectra (λ_ex_ = 484 nm for **OTK-2** [33] and 512 nm for **OTT-2**) of **OTK-2** and **OTT-2** in the solid state. Insets in (a) and (b): color and fluorescence images of **OTK-2** (left) and **OTT-2** (right) in toluene. Insets in (c) and (d): color and fluorescence images of **OTK-2** (left) and **OTT-2** (right) in the solid state. The photos depicted as insets in Figure 1a–d were reproduced from [33] (“Mechanofluorochromism of (D–π–)_2_A-type azine-based fluorescent dyes, © 2022 K. Takemura et al., published by the Royal Society of Chemistry, distributed under the terms of the Creative Commons Attribution 3.0 Unported License, https://creativecommons.org/licenses/by/3.0/).

The photoabsorption and fluorescence spectra of **OTK-2** and **OTT-2** in toluene are shown in Figure 1a,b, and their optical data are summarized in Table 1. As shown in insets of Figure 1a,b, the toluene solutions of **OTK-2** and **OTT-2** are nearly-colorless and greenish-yellow, and show blue and green fluorescent colors, respectively. The photoabsorption spectra demonstrate that the photoabsorption maximum wavelength (λ_max, abs_ = 424 nm) of **OTT-2** occurs at a by 29 nm longer wavelength than that (λ_max, abs_ = 395 nm) of **OTK-2**. The ε_max_ value for the λ_max, abs_ of **OTT-2** is 89 200 M^−1^ cm^−1^, which is comparable to that (ε_max_ = 98 000 M^−1^ cm^−1^) of **OTK-2**. In the corresponding fluorescence spectra, as in the case of **OTK-2**, **OTT-2** exhibited a vibronically-structured fluorescence band. The fluorescence maximum (λ_max,fl_) of **OTT-2** appeared at 490 nm, which is a by 43 nm longer wavelength than that (λ_max,fl_ = 447 nm) of **OTK-2**. The Stokes shift (SS) value of **OTT-2** is estimated to be 3177 cm^−1^, which is higher than that (2945 cm^−1^) of **OTK-2**. In addition, the Φ_fl_ of **OTT-2** is 0.41, which is higher than that (Φ_fl_ = 0.36) of **OTK-2**. Time-resolved fluorescence spectroscopy of the two dyes revealed that the fluorescence lifetimes (τ_fl_) are 0.62 ns for **OTK-2** and 0.66 ns for **OTT-2**, indicating that there is a little difference in the τ_fl_ values of the two dyes. The radiative rate constant (*k*_r_ = 6.2 × 10^8^ s^−1^) for **OTT-2** is slightly larger than that (5.8 × 10^8^ s^−1^) for **OTK-2**. However, the nonradiative rate constant (*k*_nr_ = 8.9 × 10^8^ s^−1^) of **OTT-2** is smaller than that (1.0 × 10^9^ s^−1^) for **OTK-2**. As the result, the ratio of nonradiative constant to radiative constant (*k*_nr_/*k*_r_ = 1.4) for **OTT-2** is smaller than that (1.7) for **OTK-2**, suggesting that the higher Φ_fl_ value of **OTT-2** is mainly attributed to the smaller *k*_nr_ value compared with that of **OTK-2**.

**Table 1:** Optical data of **OTK-2** [33] and **OTT-2** in toluene.

Dye	λ_max,abs_ [nm] (ε [M^−1^cm^−1^])	λ_max,fl_ [nm] (Φ_f_)^a^	SS [cm^−1^]^b^	τ_fl_ [ns]^c^	*k*_r_ [s^−1^]^d^	*k*_nr_ [s^−1^]^e^	*k*_nr_/*k*_r_

**OTK-2**	395 (98 000)	447 (0.36)	2945	0.62	5.8 × 10^8^	1.0 × 10^9^	1.7
**OTT-2**	424 (89 200)	490 (0.41)	3177	0.66	6.2 × 10^8^	8.9 × 10^8^	1.4

^a^Fluorescence quantum yields (Φ_fl_) were determined by using a calibrated integrating sphere system (λ_ex_ = λ_max,abs_); ^b^Stokes shift; ^c^fluorescence lifetime; ^d^radiative rate constant (*k*_r_ = Φ_fl_/τ_fl_); *^e^*nonradiative rate constant (*k*_nr_ = (1 − Φ_fl_)/τ_fl_).

The solid-state optical properties of **OTK-2** and **OTT-2** were investigated by solid-state UV–vis diffuse reflection–photoabsorption and fluorescence spectral measurements, and time-resolved fluorescence spectroscopy for the solids (Figure 1c,d). As shown in insets of Figure 1c,d, in the solid state, the colors are yellowish orange for **OTK-2** and orange for **OTT-2**, and the fluorescent colors are greenish yellow for **OTK-2** and yellow for **OTT-2**. The photoabsorption bands of **OTK-2** and **OTT-2** in the solid state are broadened in a longer wavelength region with an onset of ca. 520–550 nm, and the λ_max,abs-solid_ of **OTK-2** and **OTT-2** appeared at around 480 nm, which showed bathochromic shifts by 85 nm and 56 nm, respectively, compared with those in toluene (Table 2). The corresponding solid-state fluorescence spectra demonstrated that as in the case of toluene solutions, **OTK-2** and **OTT-2** in the solid state exhibited a vibronically-structured fluorescence band. The λ_max,fl-solid_ of **OTK-2** and **OTT-2** occur at 543 nm and 552 nm, which exhibited significant bathochromic shifts of 96 nm and 62 nm, respectively, compared with those in toluene. The Φ_fl-solid_ (0.24) of **OTT-2** is higher than that (Φ_fl-solid_ = 0.15) of **OTK-2**, while the Φ_fl-solid_ of **OTK-2** and **OTT-2** are lower than those in toluene. Although single crystals of **OTK-2** and **OTT-2** with sufficient size for X-ray structural analysis were not obtained, the intermolecular π–π interactions between the fluorophores leading to delocalization of excitons or excimers in the solid state would be responsible for the bathochromic shifts of λ_max,abs_ and λ_max,fl_ and lowering of Φ_fl_ with change of state from solution to solid [34–36]. The τ_fl-solid_ values of **OTK-2** and **OTT-2** are longer than those in toluene, however, the τ_fl-solid_ value (1.03 ns) of **OTT-2** is comparable to that (τ_fl-solid_ = 0.93 ns) of **OTK-2**. Whereas the *k*_r-solid_ value (2.3 × 10^8^ s^−1^) for **OTT-2** is larger than that (1.6 × 10^8^ s^−1^) for **OTK-2**, the *k*_nr-solid_ value (7.4 × 10^8^ s^−1^) for **OTT-2** is slightly smaller than that (9.4 × 10^8^ s^−1^) for **OTK-2**. Consequently, the *k*_nr-solid_/*k*_r-solid_ values for **OTK-2** and **OTT-2** in the solid state are 5.7 and 3.2, respectively, which are larger than those (1.7 and 1.4, respectively) in toluene, indicating that the non-radiative decay in the solid state is accelerated. However, the *k*_nr-solid_/*k*_r-solid_ value (3.2) of **OTT-2** is smaller than that (5.7) of **OTK-2**, suggesting that the higher Φ_fl-solid_ value of **OTT-2** is due to the larger *k*_r_ value compared with that of **OTK-2**. Therefore, it was found that the (D–π)_2_-type structure exhibits not only the bathochromic shift of photoabsorption band but also intense fluorescence emission both in solution and the solid state, compared to the (D–π)_2_Ph-type structure.

**Table 2:** Optical data of **OTK-2** [33] and **OTT-2** in the solid-state.

Dye	λ_max,abs-solid_ [nm]	λ_max,fl-solid_ [nm] (Φ_fl-solid_)^a^	τ_fl-solid_ [ns]^b^	*k*_r-solid_ [s^−1^]^c^	*k*_nr-solid_ [s^−1^]^d^	*k*_nr-solid_ */k*_r-solid_

**OTK-2**	480^shoulder^	543 (0.15)	0.93	1.6 × 10^8^	9.1 × 10^8^	5.7
**OTT-2**	480^shoulder^	552 (0.24)	1.03	2.3 × 10^8^	7.4 × 10^8^	3.2

^a^Fluorescence quantum yields (Φ_fl-solid_) were determined by using a calibrated integrating sphere system (484 nm for **OTK-2** and λ_ex_ = 512 nm for **OTT-2**, respectively); ^b^fluorescence lifetime; ^c^radiative rate constant (*k*_r-solid_ = Φ_fl-solid_/τ_fl-solid_); ^d^nonradiative rate constant (*k*_nr-solid_ = (1 − Φ_fl-solid_)/τ_fl-solid_).

**Figure 2:**
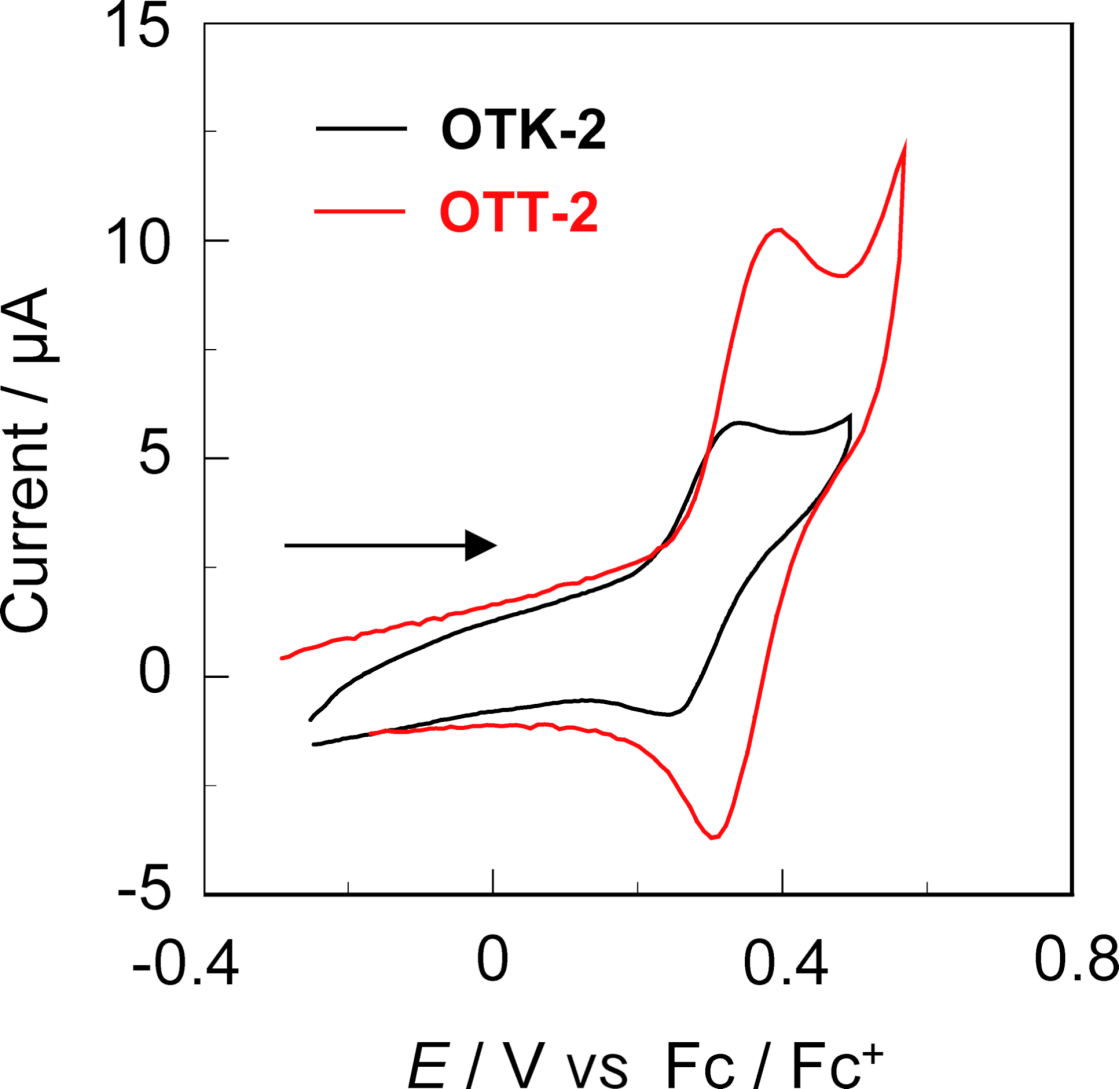
Cyclic voltammograms of **OTK-2** and **OTT-2** (0.1 mM) in DMF containing 0.1 M Bu_4_NClO_4_ at a scan rate of 100 mV s^−1^. The arrow denotes the direction of the potential scan.

**Figure 3:**
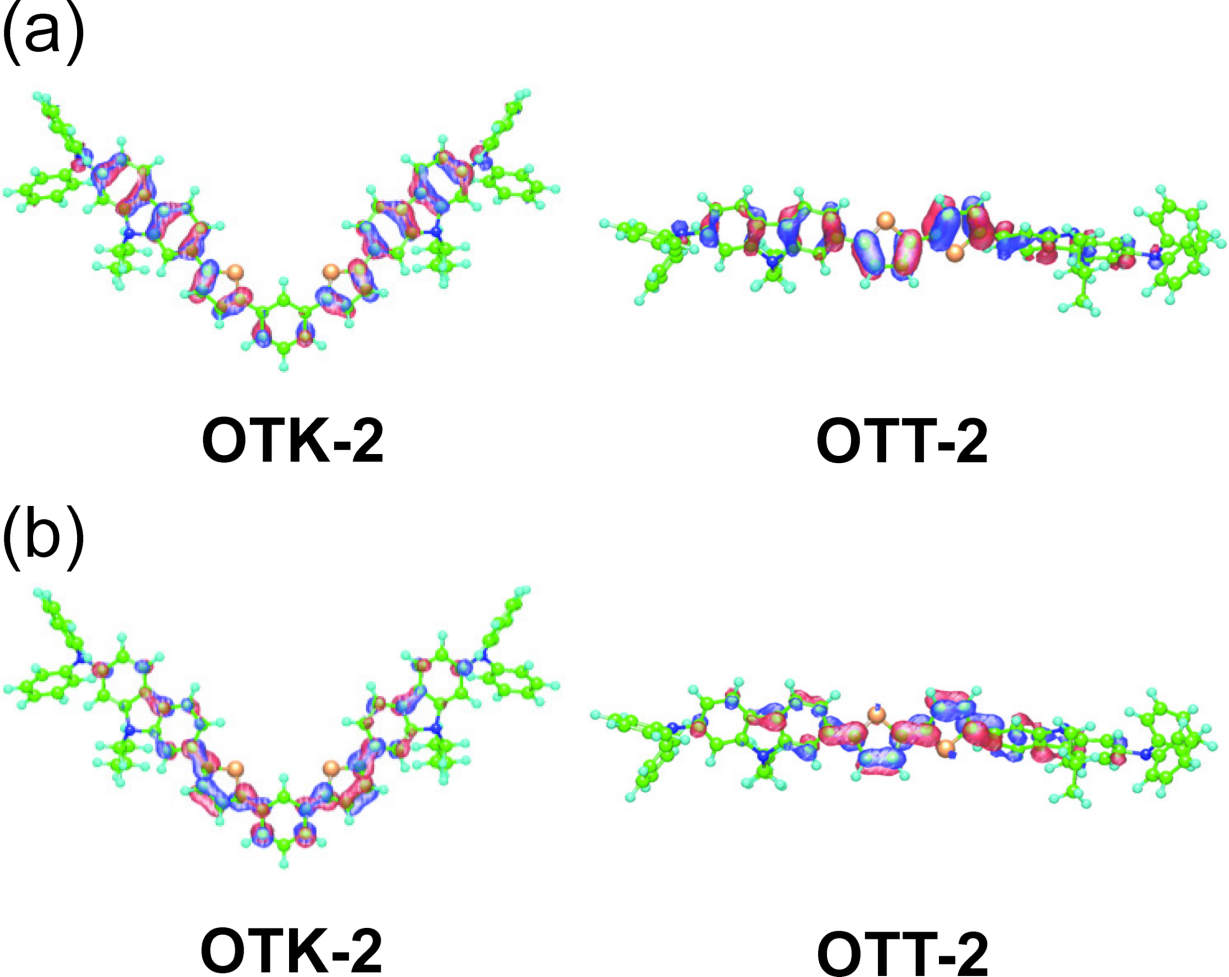
(a) HOMO and (b) LUMO of **OTK-2** [33] and **OTT-2** derived from MO calculations (PM5, INDO/S method). The red and blue lobes denote the positive and negative signs of the coefficients of the molecular orbitals. The size of each lobe is proportional to the MO coefficient.

The electrochemical properties of **OTK-2** and **OTT-2** (0.1 mM) were evaluated using CV in DMF containing 0.1 M tetrabutylammonium perchlorate (Bu_4_NClO_4_), in which the potentials were internally referenced to ferrocene/ferrocenium (Fc/Fc^+^). The electrochemical data are summarized in Table 3. The cyclic voltammograms of the two dyes show a reversible oxidation wave with the anodic peak potential (*E*_pa_^ox^) at 0.32 V for **OTK-2** and 0.40 V for **OTT-2** (Figure 2), while any obvious reduction waves and another oxidation waves did not appear within the potential window (Figure 3a and Figure S2a, Supporting Information File 1). The corresponding cathodic peak potential (*E*_pc_^ox^) appeared at 0.24 V for **OTK-2** and 0.30 V for **OTT-2**, and thus the peak separations between the *E*_pa_^ox^ and *E*_pc_^ox^ waves are ca. 80–100 mV. This result may indicate that the two dyes undergo an electrochemically stable one-electron oxidation–reduction process, but further studies are necessary to exactly determine the number of electrons in the oxidation–reduction process. The half-wave potential (*E*_1/2_^ox^) was evaluated to be 0.28 V for **OTK-2** and 0.35 V for **OTT-2**. Therefore, the *E*_1/2_^ox^ for **OTK-2** with the (D–π)_2_Ph-type structure is cathodically shifted by 0.07 V, compared with that for **OTT-2** with the (D–π)_2_-type structure. Furthermore, we investigated the diffusion-controlled process form CV at different scan rates (50, 100, 200, 400, 600 and 1000 mV s^−1^) and reversibility of the oxidation process by repeated potential cycling (20 cycles). For both **OTK-2** and **OTT-2**, the *E*_pa_^ox^ remained steady at different scan rates while the anodic peak current (*I*_pa_) increased with the increase in scan rate. The *I*_pa_ showed a negligible change during 20 cycles at a scan rate of 100 mV s^−1^, indicating diffusion control and good reversibility of the oxidation process (Figures S2b,c and S3b,c, Supporting Information File 1). The highest occupied molecular orbital (HOMO) energy level versus vacuum level was estimated from the *E*_1/2_^ox^, that is, −[*E*_1/2_^ox^ + 4.8] eV. On the other hand, the lowest unoccupied molecular orbital (LUMO) energy level versus the vacuum level was estimated by using [HOMO + *E*_0–0_] eV from the *E*_1/2_^ox^ and intersections (optical energy gap: *E*_0-0_ = 2.79 eV for **OTK-2** and 2.61 eV for **OTT-2**) of the photoabsorption and fluorescence spectra in toluene. It was found that the HOMO energy level (−5.15 eV) of **OTT-2** is slightly lower than that (−5.08 eV) of **OTK-2**, indicating that the two dyes have comparable HOMO energy levels. On the other hand, the LUMO energy level (−2.54 eV) of **OTT-2** is significantly lower than that (−2.29 eV) of **OTK-2**. Semi-empirical MO calculations (PM5, INDO/S method) revealed that for **OTK-2** both the HOMO and LUMO were mostly localized on the two (diphenylamino)carbazole-thiophene moieties. On the other hand, for **OTT-2** both the HOMO and LUMO are delocalized over the whole molecule through the thiophene units (Figure 3). Consequently, the fact reveals that compared to the (D–π)_2_Ph-type structure, the (D–π)_2_-type structure can cause not only the stabilization of the LUMO energy level but also the delocalization of the HOMO and LUMO over the whole molecule, leading to a narrower HOMO–LUMO band gap of **OTT-2** than **OTK-2**, that is, the bathochromic shift of the photoabsorption band from **OTT-2** to **OTK-2**.

**Table 3:** Electrochemical data, and HOMO and LUMO energy levels of **OTK-2** and **OTT-2**.

Dye	*E*_pa_^ox^ [V]^a^	*E*_pc_^ox^ [V]^a^	*E*_1/2_^ox^ [V]^a^	HOMO [eV]^b^	LUMO [eV]^c^	*E*_0–0_ [eV]^d^

**OTK-2**	0.32	0.24	0.28	−5.08	−2.29	2.79 eV
**OTT-2**	0.40	0.30	0.35	−5.15	−2.54	2.61 eV

^a^The anodic peak (*E*_pa_^ox^), the cathodic peak (*E*_pc_^ox^) and the half-wave (*E*_1/2_^ox^) potentials for oxidation vs Fc/Fc^+^ were recorded in DMF/Bu_4_NClO_4_ (0.1 M) solution; ^b^−[*E*^ox^_1/2_ + 4.8] eV; ^c^[HOMO + *E*_0–0_] eV; ^d^444 nm for **OTK-2** and 475 nm for **OTT-2**.

## Conclusion

We have developed the (D–π)_2_-type fluorescent dye **OTT-2** and the (D–π)_2_Ph-type fluorescent dye **OTK-2** and evaluated their optical and electrochemical properties. Both in solution and the solid state, the photoabsorption and fluorescence maximum wavelengths of **OTT-2** appear in a longer wavelength region than those of **OTK-2**, and the fluorescence quantum yields of **OTT-2** are higher than those of **OTK-2**. The cyclic voltammograms demonstrated that **OTT-2** and **OTK-2** exhibit a reversible oxidation wave, indicating that the two dyes undergo an electrochemically stable oxidation–reduction process. It was found that the LUMO energy level of **OTT-2** is lower than that of **OTK-2**, while **OTT-2** and **OTK-2** have comparable HOMO energy levels. Semi-empirical MO calculations showed that for **OTK-2** both the HOMO and LUMO were mostly localized on the two D–π moieties, whereas for **OTK-2** both the HOMO and LUMO are delocalized over the whole molecule through the thiophene units. Consequently, this work reveals that compared to the (D–π)_2_Ph-type structure, the (D–π)_2_-type structure not only has intense fluorescence emission properties both in solution and the solid state, but also can cause delocalization of the HOMO and the LUMO over the whole molecule as well as the stabilization of the LUMO energy level, leading to a narrower HOMO–LUMO band gap of **OTT-2** than **OTK-2**, that is, the bathochromic shift of photoabsorption band from **OTT-2** to **OTK-2**.

## Experimental

### General methods

Melting points were measured with an AS ONE ATM-02 apparatus. IR spectra were recorded on a SHIMADZU IRTracer-100 spectrometer by ATR method. ^1^H NMR and ^13^C NMR spectra were recorded on a Varian-500 FT NMR spectrometer. High-resolution mass spectral data by APCI were acquired on a Thermo Fisher Scientific LTQ Orbitrap XL apparatus. Photoabsorption spectra of solutions were observed with a Shimadzu UV-3600 plus spectrophotometer. Photoabsorption spectra of solids were recorded by a Shimadzu UV-3600 plus spectrophotometer with a calibrated integrating sphere system. Fluorescence spectra of solutions and solids were measured with a HORIBA FluoroMax-4 spectrofluorometer. Fluorescence quantum yields in solution and in the solid state were determined using a HORIBA FluoroMax-4 spectrofluorometer with a calibrated integrating sphere system. Fluorescence decay measurements were performed on a HORIBA DeltaFlex modular fluorescence lifetime system using a Nano LED pulsed diode excitation source (451 nm). Cyclic voltammetry (CV) curves were recorded in DMF/Bu_4_NClO_4_ (0.1 M) solution with a three-electrode system consisting of Ag/Ag^+^ as the reference electrode, a Pt plate as the working electrode and a Pt wire as the counter electrode using an Electrochemical Measurement System HZ-7000 (HOKUTO DENKO). Semi-empirical molecular orbital calculations were carried out with the WinMOPAC Ver. 3.9 package (Fujitsu, Chiba, Japan), where geometry calculations of the compounds in the ground state were made using the PM5 method. Dipole moments and HOMO and LUMO energy levels of the compounds were also evaluated from INDO/S calculations.

### Synthesis

**7,7'-(1,3-Phenylenebis(thiophene-5,2-diyl))bis(9-butyl-*N*,*N*-diphenyl-9*H*-carbazol-2-amine) (OTK-2) and 7,7'-([2,2'-bithiophene]-5,5'-diyl)bis(9-butyl-*N*,*N*-diphenyl-9*H*-carbazol-2-amine) (OTT-2):** A solution of **1** (87 mg, 0.137 mmol), 1,3-diiodobenzene (14 mg, 0.041 mmol), and Pd(PPh_3_)_4_ (2 mg, 0.001 mmol) in toluene (1 mL) was stirred for 29 h at 110 ºC under an argon atmosphere. The reaction mixture was diluted with water, and then, the solution was extracted with dichloromethane. The dichloromethane extract was dried over anhydrous MgSO_4_, filtrated, and concentrated. The residue was chromatographed on silica gel (ethyl acetate/hexane 1:4 ) to give **OTK**-**2** (19 mg, yield 27%) and **OTT-2** (9 mg, yield 14%) as a light yellow solid and an orange solid, respectively; the characterization data for **OTK-2** are in agreement with those reported in the literature [33]; **OTT-2**: mp >300 °C; FTIR (ATR) ν̄: 1591, 1491, 1460 cm^−1^; ^1^H NMR (500 MHz, CD_2_Cl_2_) δ 0.76–1.02 (m, 6H), 1.23–1.38 (m, 4H), 1.71–1.81 (m, 4H), 4.12–4.21 (m, 4H), 6.95 (dd, *J* = 1.8 and 8.4 Hz, 2H), 7.01–7.05 (m, 4H), 7.10–7.16 (m, 10H), 7.24–7.31 (m, 10H), 7.39 (d, *J* = 3.8 Hz, 2H), 7.50 (dd, *J* = 1.4 and 8.0 Hz, 2H), 7.59 (d, *J* = 1.2 Hz, 2H), 7.93 (d, *J* = 8.3 Hz, 2H), 7.99 (d, *J* = 8.0 Hz, 2H) ppm; ^13^C NMR (125 MHz, CD_2_Cl_2_) δ 14.04, 20.85, 31.44, 43.04, 105.37, 105.84, 117.54, 117.56, 118.73, 120.46, 121.16, 122.94, 122.96, 124.02, 124.36, 124.84, 129.55, 131.14, 136.68, 141.67, 142.73, 144.74, 146.92, 148.61 ppm; HRMS (APCI) *m*/*z* (%): [M + H^+^] calcd. for C_64_H_55_N_4_S_2_, 943.38627; found, 943.38635.

## Supporting Information

File 1 ^1^H and ^13^C NMR spectra of **OTT-2**.
