# Supplementary material for: Synthesis, optical and electrochemical properties of (D–π)2-type and (D–π)2Ph-type fluorescent dyes
Source: Beilstein J Org Chem. 2022 Aug 18;18:1047–54. doi: 10.3762/bjoc.18.106 (PMC9443425; doi:10.3762/bjoc.18.106)
Supplement: File 1 — 1H and 13C NMR spectra of OTT-2. [file Beilstein_J_Org_Chem-18-1047-s001.pdf]

BEILSTEIN JOURNAL OF ORGANIC CHEMISTRY

Supporting Information

for

Synthesis, optical and electrochemical properties of $(D–\pi)_2$-type and $(D–\pi)_2$Ph-type fluorescent dyes

Kosuke Takemura, Kazuki Ohira, Taiki Higashino, Keiichi Imato and Yousuke Ooyama

Beilstein J. Org. Chem. **2022,** *18,* 1047–1054. doi:10.3762/bjoc.18.106

^{1}H and ^{13}C NMR spectra of OTT-2

License and Terms: This is a supporting information file under the terms of the Creative Commons Attribution License (https://creativecommons.org/licenses/by/4.0). Please note that the reuse, redistribution and reproduction in particular requires that the author(s) and source are credited and that individual graphics may be subject to special legal provisions.
The license is subject to the *Beilstein Journal of Organic Chemistry* terms and conditions: (https://www.beilstein-journals.org/bjoc/terms)

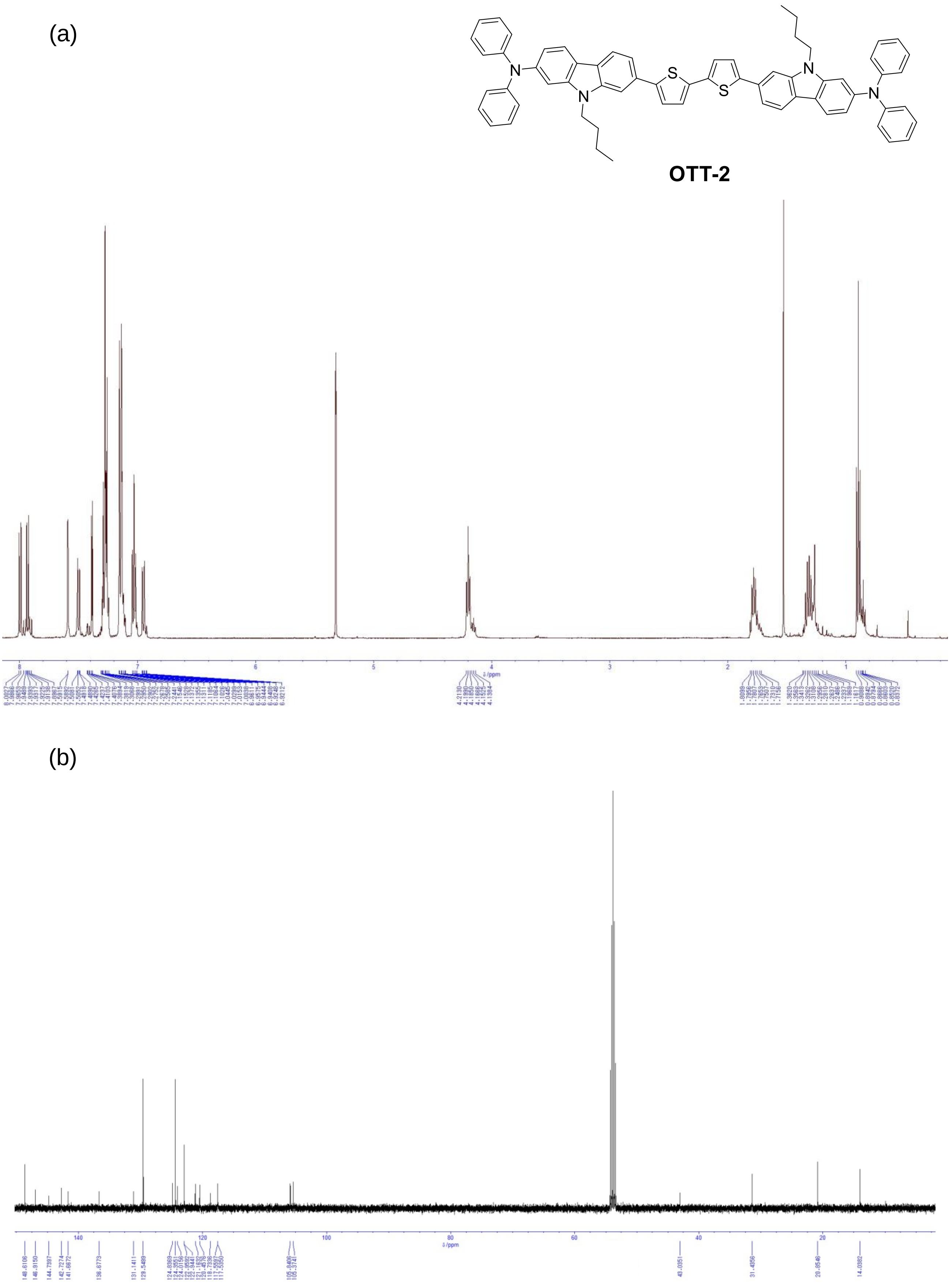

Figure S1: (a) ^{1}H HMR (500 MHz) and (b) ^{13}C HMR (125 MHz) spectra of **OTT-2** in CD_2Cl_2.

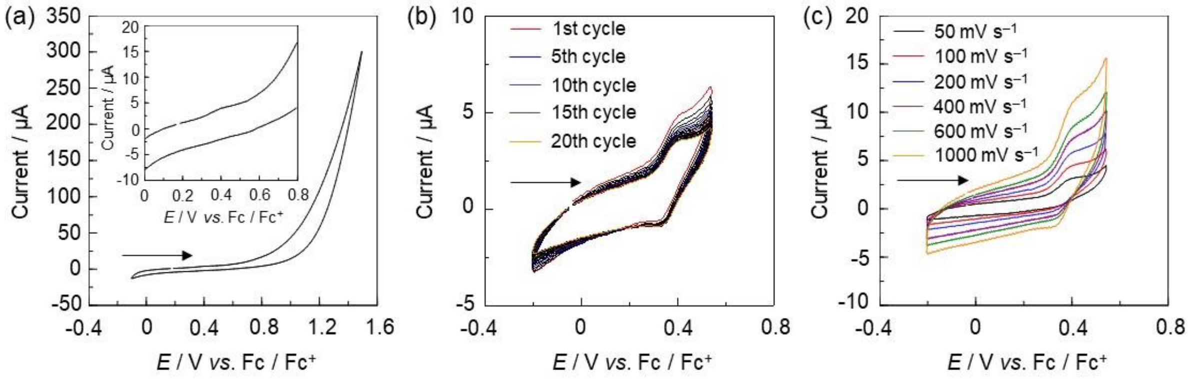

Figure S2: Cyclic voltammograms of **OTK-2** (0.1 mM) in DMF containing 0.1 M Bu_4NClO_4 (a) at a scan rate of 100 mV s^{-1} in a broader potential range (−0.2 to 1.5 V), (b) by the repeated potential cycling (20th scan) at a scan rate of 100 mV s^{-1} and (c) at different scan rates (50, 100, 200, 400, 600 and 1000 mV s^{-1}). The arrow denotes the direction of the potential scan. The inset in (a) is magnification of potential range from 0 to 0.8 V.

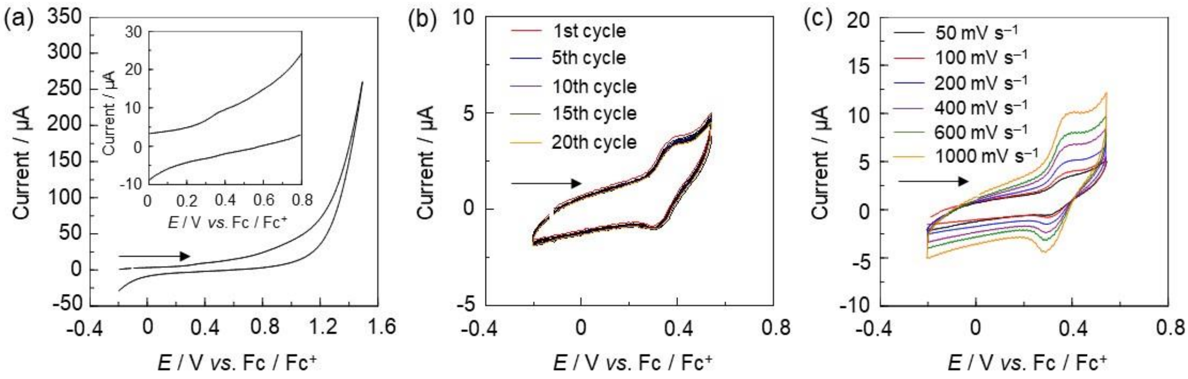

Figure S3: Cyclic voltammograms of **OTT-2** (0.1 mM) in DMF containing 0.1 M Bu_4NClO_4 (a) at a scan rate of 100 mV s^{-1} in a broader potential range (−0.2 to 1.5 V), (b) by the repeated potential cycling (20th scan) at a scan rate of 100 mV s^{-1} and (c) at different scan rates (50, 100, 200, 400, 600 and 1000 mV s^{-1}). The arrow denotes the direction of the potential scan. The inset in (a) is magnification of potential range from 0 to 0.8 V.
